# Automated Hypothesis Generation to Identify Signals Relevant in the Development of Mammalian Cell and Tissue Bioprocesses, With Validation in a Retinal Culture System

**DOI:** 10.3389/fbioe.2020.00534

**Published:** 2020-06-04

**Authors:** Derek Toms, Abdullah Al-Ani, Saud Sunba, Qing Yun (Victor) Tong, Matthew Workentine, Mark Ungrin

**Affiliations:** ^1^Department of Comparative Biology and Experimental Medicine, Faculty of Veterinary Medicine, University of Calgary, Calgary, AB, Canada; ^2^Alberta Children’s Hospital Research Institute, University of Calgary, Calgary, AB, Canada; ^3^Biomedical Engineering Graduate Program, University of Calgary, Calgary, AB, Canada; ^4^Alberta Diabetes Institute, University of Alberta, Edmonton, AB, Canada; ^5^Leaders in Medicine Program, Cumming School of Medicine, University of Calgary, Calgary, AB, Canada

**Keywords:** bioinformatics, microarray, code:R, receptor, signaling, retina

## Abstract

We have developed an accessible software tool (receptoR) to predict potentially active signaling pathways in one or more cell type(s) of interest from publicly available transcriptome data. As proof-of-concept, we applied it to mouse photoreceptors, yielding the previously untested hypothesis that activin signaling pathways are active in these cells. Expression of the type 2 activin receptor (*Acvr2a*) was experimentally confirmed by both RT-qPCR and immunochemistry, and activation of this signaling pathway with recombinant activin A significantly enhanced the survival of magnetically sorted photoreceptors in culture. Taken together, we demonstrate that our approach can be easily used to mine publicly available transcriptome data and generate hypotheses around receptor expression that can be used to identify novel signaling pathways in specific cell types of interest. We anticipate that receptoR (available at https://www.ucalgary.ca/ungrinlab/receptoR) will enable more efficient use of limited research resources.

## Introduction

The ability to understand and manipulate cellular behavior is critical to conventional small-molecule pharmaceutical therapies as well as the rapidly growing fields of tissue engineering, regenerative medicine and cell-based therapy (Alimbetov et al., 2018; Dalby et al., 2018; Loebel and Burdick, 2018; Zhang et al., 2018; Manoukian et al., 2019). Aside from the direct manipulation of transcription factors, much of our capacity to control this behavior comes via intervention in cellular signaling cascades. In this way, the expansion of human pluripotent stem cells (hPSCs) may be enhanced (Lipsitz et al., 2018) and their differentiation directed to fates as diverse as retinal pigment epithelial cells (da Cruz et al., 2018) or insulin producing beta cells (Rezania et al., 2014). Chimeric antigen receptor (CAR) T cells function via an engineered signaling cascade that can redirect T cells toward a specific antigen (reviewed in Jackson et al., 2016), while cytokine traps that remove targeted ligands (Economides et al., 2003) have shown promise as treatments for macular edema caused by an overgrowth of endothelial cells (Heier et al., 2012). These diverse applications share the common mechanism of intervening in pre-existing signaling pathways within the relevant cell types.

While pathways that are active in a given type of cell may be inferred from previous developmental or functional studies (Zhou et al., 2015; Fan et al., 2016; Yoon et al., 2018; van der Kant et al., 2019), such research may not yet have been completed for a given tissue of interest. Even if it has, there is no guarantee that all relevant interactions have been identified. While efforts such as the Human Cell Atlas (Rozenblatt-Rosen et al., 2017) promise to facilitate data sharing, it is challenging for researchers who have identified a role for a particular cell type in their disease of interest, to enter new areas and acquire the depth of specialist knowledge required to predict potential interventions.

In attempting to manipulate the behavior of a given cell type, an important starting point is simply “What receptors does this cell express?” There is no point in adding a particular factor to the culture medium if the cell lacks the machinery required to respond to it (Miyajima et al., 1992; Krebs and Hilton, 2000; Uings and Farrow, 2000). Conversely, while expression of a certain receptor does not guarantee its downstream signaling function (Ris-Stalpers et al., 1990; Robbins et al., 1993), it does immediately provide us with a pair of easily testable hypotheses: firstly, that *the cell will respond to its activation* in some way; and secondly that *this response already occurs* at some point within the range of environments to which that cell type is normally exposed (niche) – and potentially in the culture system of interest as well. Additional knowledge about the function of that signaling pathway in other contexts may be informative (Cai et al., 2016; Harskamp et al., 2016; Schwartz et al., 2016; Pavlos and Friedman, 2017) but is not required (and is not necessarily complete in any case) (Cendrowski et al., 2016). The cells may then be exposed to activators and inhibitors of the receptor identified, and the impact assessed. Should a response be observed (for example, on function or proliferation), if it is a desirable one then the ligand concentration can be optimized and routinely incorporated into the bioprocess under development; if it is undesirable then antagonism of that pathway may similarly be employed.

Where resources are available, receptor expression may be characterized specifically in a system of interest. However, funding agencies are often unwilling to support expensive “fishing expeditions” and even if funding is available, it could be employed elsewhere if less expensive approaches could be identified. We therefore sought to automate the generation of hypotheses about the presence and function of receptors using the significant quantity of existing gene transcript resources. While increasingly ceding ground to RNA sequencing (RNA-seq) approaches, over half the data series available on the publicly-accessible Gene Expression Omnibus (GEO) database derive from expression profiling by microarray, with high throughput sequencing platforms making up less than one quarter. Furthermore, both technologies offer high throughput assessment of gene expression with similar quantitation accuracy and high technical reproducibility (reviewed in Lowe et al., 2017). Despite a more limited dynamic range and lower sensitivity than either RNA-seq or quantitative PCR, microarrays have proven to be a reliable technology for detecting significantly enriched genes between tissue types, and robust expression patterns between all three technologies correlate well (Larkin et al., 2005; Allanach et al., 2008; Marioni et al., 2008; Su et al., 2014). Microarray data has been collected on platforms with a high degree of uniformity, and for which minimum information standards already exist (Brazma et al., 2001; Ball et al., 2004). Originally obtained to test specific hypotheses, in aggregate they contain a tremendous amount of information on, e.g., untreated control groups that may not otherwise have been previously assessed.

The most straight-forward way to access these data are with open source tools that can query the GEO database (Gentleman et al., 2004; Davis and Meltzer, 2007; R Development Core Team, 2008; Huber et al., 2015), however these typically make use of the command line and can have a steep learning curve. Several bioinformatics tools have been developed to provide a graphical user interface to this data (Dumas et al., 2016; Nelson et al., 2017), although these are limited to analyzing a single experimental series at once. We therefore developed a software tool – receptoR – to enable non-bioinformaticians to access and then aggregate this data, and rapidly generate hypotheses about signaling pathways that may be relevant to the cell and tissue types they are studying. We validated receptoR’s performance in retinal photoreceptor cells, as their survival and function *in vitro* and *in vivo* are highly influenced by cytokines derived from their niche (Strauss, 2005; Jindal, 2015). As the first component of our visual pathway they are essential for sight and therefore hugely impactful on human health and quality of life.

In the United States alone, visual impairment not due to a refractive error affects 2% of the adult population, over six million people, with associated costs exceeding $5.5 billion annually (Congdon et al., 2004; Frick et al., 2007; Chou et al., 2013). When looking at age-related macular degeneration (AMD) specifically, the most common cause of visual dysfunction in industrialized countries, incidence increases to 20% of people over 65 years of age (Mitchell et al., 1995; Vingerling et al., 1995; Huang et al., 2003; Kashani et al., 2018). Absent injury or infection of the retina, most visual dysfunction qualifies as inherited retinal degeneration, a genetically heterogeneous group of disorders affecting the viability and function of rod and cone photoreceptors that can have autosomal, X-linked, and mitochondrial patterns of inheritance (Farrar et al., 2015; Thompson et al., 2015). Over 200 causative genes have been identified that affect multiple pathways and mechanisms associated with vision dysfunctions (Thompson et al., 2015). Retinal degenerative diseases can also be a consequence of genetic dysfunction in the underlying retinal pigment epithelium (RPE) or vasculature that support the retina (Bhutto and Lutty, 2012; Alexander et al., 2015; Farrar et al., 2015). Given the complexity and scope of the underlying causes, curative treatments are not currently available, with most clinical interventions aiming to slow the progression of the disorders (Rolling, 2004). This approach has seen some success, and studies have demonstrated attenuation of photoreceptor loss in animal models of retina degeneration using exogenous delivery of signaling molecules including pigment epithelium-derived factor (PEDF), brain-derived neurotrophic factor (BDNF), ciliary neurotrophic factor (CNTF) and several fibroblast growth factors (FGFs) (LaVail et al., 1992; Cayouette and Gravel, 1997; Caffé et al., 2001; Green et al., 2001; Liang et al., 2001; Azadi et al., 2007; Moeller and Neubert, 2013; Kimura et al., 2016; Comitato et al., 2018). Understanding signaling pathways that maintain healthy photoreceptors is therefore critical to the development of new approaches to maintain existing photoreceptor cells, as well as potentially curative future cell-based therapies to replace them (Pearson et al., 2012; Santos-Ferreira et al., 2014).

In the present study, we used our bioinformatics tool, receptoR, to identify activin receptor 2A (*Acvr2a*) as a target present in post-mitotic photoreceptors that can be activated to increase their *in vitro* survival.

## Results

**FIGURE 1:**
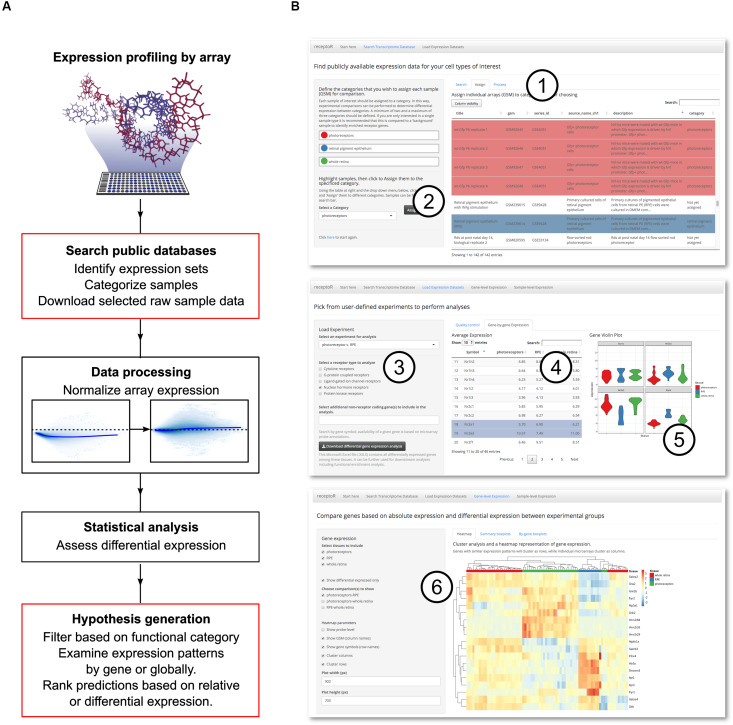
Digitized gene expression data can be mined to predict receptor pathways. Experimental microarray data is publicly available for download and reanalysis, and can be used to make informed hypotheses about cell- or tissue-specific receptor expression. **(A)** The pipeline for mining, categorizing and analyzing the microarray data. User interaction steps are outlined in red. **(B)** Our web application, receptoR, allows users to analyze microarray experiments by searching public experimental series for specific sample expression data (1) and categorizing each sample according to their experimental design (2). After retrieving and processing the expression data, predictions can be filtered by genes coding for specific receptor types (3), individual gene data can be sorted (4) and visualized (5). Absolute expression levels of receptor-coding genes can be clustered based on assigned categories and filtered based on differential expression between groups (6).

Our overall approach comprises the identification and importing of relevant datasets; normalization to allow comparisons; initial automated analysis; and finally, user-interactive analysis to identify and extract specific information of interest (Figure 1A). As we have regular access to murine retinal cells via a secondary-use ethics approval, we elected to focus on mouse transcript data, although receptoR is able to work with both human and mouse data. This pipeline was developed with the non-bioinformatician user in mind, and our web-based graphical user interface facilitates the mining of datasets from the GEO database, categorization of the retrieved samples, and downstream analysis. Results presented here thus make use of the receptoR app except where explicitly stated; details of how the data is obtained and processed can be found in Section “Bioinformatics.”

We began by acquiring appropriate data records to examine the transcriptome of photoreceptors and RPE. The GEO database was searched for GEO samples; each sample record (assigned a unique accession number beginning with ‘GSM’) is the digitized image of the microarray after sample hybridization and represents the transcriptome of a single biological sample. For clarity we will refer to these samples as ‘microarrays’ or simply ‘arrays’ throughout the text. Typically, these arrays will have been deposited as part of a larger GEO experimental data series containing up to 10s of GSM array records. This process is summarized in Figure 1B with a detailed step-by-step manual included as Supplementary Methods S1.

From these search results, we selected 78 microarrays from 15 unique series, falling into of one of three categories of interest: photoreceptors (*n* = 30), RPE (*n* = 11), or whole retina (*n* = 37) for downstream analysis. Arrays were composed of purified cell populations and isolated tissues, with a median age of 7.5 days. Assignment to these categories was based on data included in the record and associated publications (Supplementary Table S1 – this data is made available to the receptoR user during the search process). Of note, while photoreceptor maturation continues in this time window, they are consistently post-mitotic and represent a widely used model of photoreceptor behavior (MacLaren et al., 2006; Brzezinski and Reh, 2015; Unachukwu et al., 2016; Waldron et al., 2018). By pooling arrays obtained in multiple experiments across multiple laboratories into a single biological category, we enhance statistical power and reduce technical bias (‘batch effect’) to which all high throughput transcriptome data are vulnerable (Turnbull et al., 2012). Importantly, receptoR will generate a warning if all arrays in a group come from the same series, as technical and biological differences will then be confounded. After assigning each array to a category, receptoR will then download the full raw data files from the final array list from GEO. Transparently to the user (see section “Normalization and Differential Gene Expression”), array data is normalized and significant differentially expressed genes (DEGs) among groups are predicted, before this data is returned to be analyzed based both on high relative expression and differential expression among groups.

**FIGURE 2:**
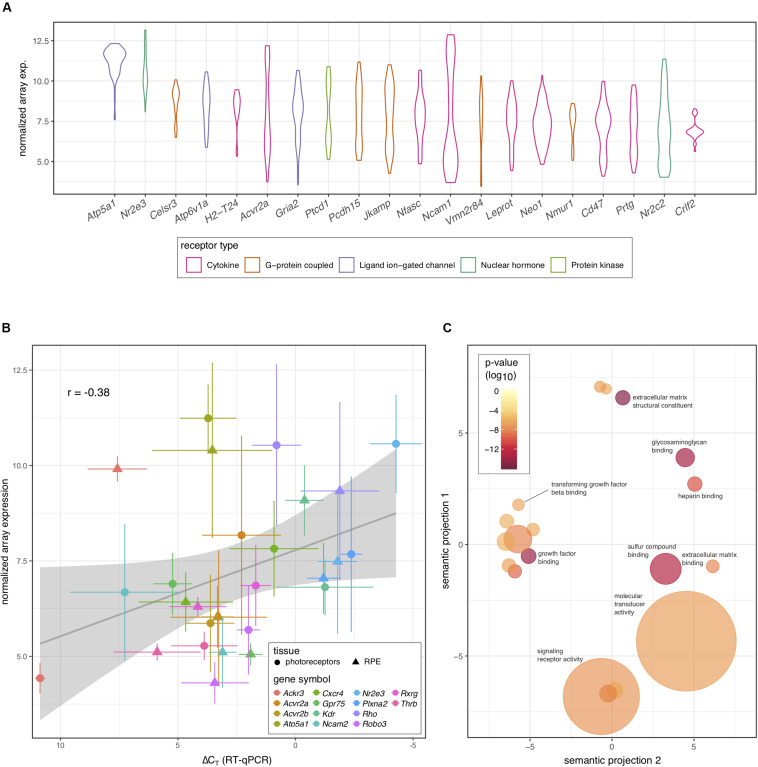
Prediction of receptor gene expression in photoreceptors. **(A)** Predicted levels of the 20 most highly expressed receptor protein-coding genes in photoreceptors that are differentially expressed between photoreceptors and RPE. Receptor type is indicated by color and plots represent the distribution of all samples (*n* = 30) across all probes. **(B)** Fourteen genes representing varying expression levels in photoreceptors and RPE were assayed using RT-qPCR (*n* = 5). Pooled archival microarray data (*n* = 30, photoreceptors; *n* = 11, RPE) showed a moderate, yet significant correlation with ΔC_*T*_ values (*R* = –0.38, *P* = 0.047). RT-qPCR expression was normalized to three endogenous control genes (*Hprt*, *Polr2a*, and *Tbp*). **(C)** Reduced gene ontology map showing photoreceptor-enriched functional categories for all DEGs between photoreceptors and RPE. Circle size is based on the frequency of the term within all UniProt annotations (smaller = less common and more specific) while the color of each circle displays the *P*-value associated with the photoreceptor enrichment of terms in that category (redder = lower). The x- and y-axes represent semantic distances between annotation keywords in arbitrary units.

Because of the known role of cytokine signaling in preventing photoreceptor degeneration (Tombran-Tink and Barnstable, 2003; Bradford et al., 2005; Dilly and Rajala, 2008) we decided to look more closely at this mode of signaling and filter those genes annotated to code for cytokine receptors. Interestingly, the activin receptor type 2A (*Acvr2a*) was predicted to be highly transcribed in photoreceptors, with comparable levels to those receptors whose ligands have been shown to be important for photoreceptor survival, including PEDF, CNTF, PDGF, IGF-1, and FGF-2 (Tombran-Tink and Barnstable, 2003; Bradford et al., 2005; Dilly and Rajala, 2008; Di Pierdomenico et al., 2018; Li et al., 2018). To our knowledge the role of activin has not been studied in this cell type. Indeed, *Acvr2a* was predicted to be the second most highly transcribed cytokine receptor in photoreceptors (Figure 2A). To validate our bioinformatic hypotheses about receptor expression in photoreceptors, 14 genes predicted to be differentially expressed between photoreceptors and RPE or highly expressed in photoreceptors (Table 1) were assayed by RT-qPCR in both tissues. When we compared the ΔC_*T*_ values against the predicted gene transcription profiles we observed a significant correlation (*P* = 0.047) (Figure 2B).

**TABLE 1:** ReceptoR prediction for highly transcribed cytokine receptors in photoreceptor and RPE.

**Gene symbol**	**photoreceptor normalized expression**	**RPE normalized expression**	**Receptor category**	**Differential expression between photoreceptors and RPE (P_*adj*_ < 0.05)**
Atp5a1	11.2	10.4	Ligand-gated ion channel	Yes
Nr2e3	10.6	7.5	Nuclear hormone	Yes
Atp5b	10.5	11.8	Ligand-gated ion channel	No
Rho	10.5	9.3	G-protein coupled	No
Cnga1	9.5	7.6	Ligand-gated ion channel	No
Cngb1	9.1	6.9	Ligand-gated ion channel	No
Rorb	8.8	7.7	Nuclear hormone	No
Celsr3	8.7	5.1	G-protein coupled	Yes
Atp6v1a	8.4	10.1	Ligand-gated ion channel	Yes
H2-T24	8.3	5.2	Cytokine	Yes
Rxrb	8.3	9.5	Nuclear hormone	No
Acvr2a	8.2	6.0	Cytokine receptor/protein kinase	Yes
Nr2f6	8.0	8.3	Nuclear hormone	No
Gria2	8.0	5.3	Ligand-gated ion channel	Yes
Ptcd1	8.0	6.0	Protein kinase	Yes
Shroom2	8.0	7.5	Ligand-gated ion channel	No
Pcdh15	8.0	4.5	G-protein coupled	Yes
Hcn1	7.9	6.7	Ligand-gated ion channel	No
Ackr4	7.9	6.5	Cytokine receptor/G-protein coupled	No
Jkamp	7.8	8.8	G-protein coupled	Yes
Cxcr4	7.8	6.4	Cytokine	No
Pcdhb20	7.8	7.3	G-protein coupled	No
Nr1d2	7.8	8.5	Nuclear hormone	No
Nfasc	7.8	6.3	Cytokine	Yes
Grm4	7.8	6.4	G-protein coupled	No
Gpr75	6.9	5.1	G-protein coupled	Yes
Ncam2	6.7	5.1	Cytokine	Yes
Ccr9	5.8	4.2	Cytokine	Yes

To further explore our prediction that activin signaling may play an important role in photoreceptors, we exported the list of all predicted DEGs between photoreceptors and RPE from receptoR (Figure 1B and Supplementary Table S2). Then, we annotated each transcript on the list to a gene ontology term to identify enriched pathways based on a ranked list of significant DEGs (adjusted *P* < 0.05; highest expression to lowest) subtracted from background transcription in the mouse (Eden et al., 2007, 2009). What we observed was a significant enrichment in transcripts coding for growth factor binding and extracellular compound binding proteins, including those binding transforming growth factor beta (TGF β; Figure 2C). This finding is consistent with photoreceptors’ role in receiving supportive signals from the RPE and with the composition of the interphotoreceptor matrix to which photoreceptors bind (Strauss, 2005; Ishikawa et al., 2015). Activin is a member of the TGF β superfamily, whose signaling is known to play a role in cell survival and growth (Chen et al., 2002), and enrichment of this pathway supported the high predicted levels of *Acvr2a* in photoreceptors.

**FIGURE 3:**
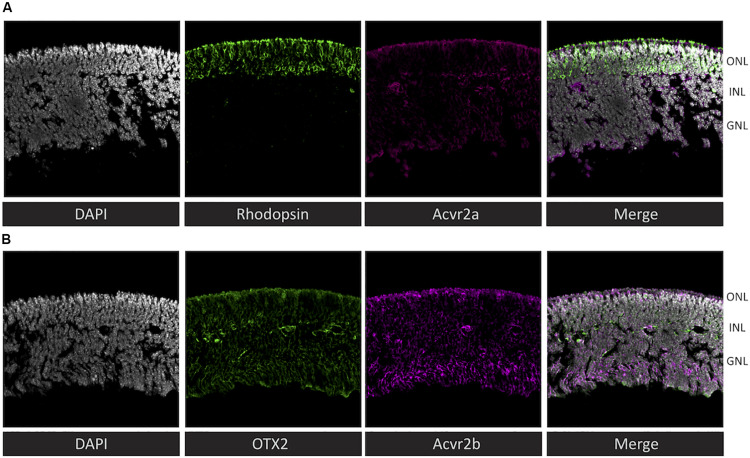
Immunohistochemistry reveals that both predicted activin receptors (Acvr2a and Acvr2b) are expressed in photoreceptors. To validate the cytokine receptor prediction by receptoR, sections of post-natal day 4 mouse retina (without RPE) were immunostained for activin type 2 receptors and well-known photoreceptor/retina markers. **(A)** Co-localization of rhodopsin (green) and Acvr2a (magenta) indicates the expression of this activin receptor in photoreceptor cells. **(B)** In contrast, Acvr2b (green) shows weaker, less specific staining throughout the retina, similar to the expression of Otx2 (magenta). Sections are 20 μm thick and were counterstained with DAPI (gray).

To assess the plausibility of the hypothetical activin signaling implied by the receptoR predictions, we examined the expression of type 2 activin receptors at the protein level in the mouse retina at post-natal day 4. We detected Acvr2a throughout the retina, with a particularly intense staining in the outer region of rhodopsin-positive cells (Figure 3A) – interestingly this is the region immediately adjacent to the RPE, which is a source for many photoreceptor-supportive signals. Acvr2b showed a similar staining pattern, with strong staining at the photoreceptor-RPE margin, as well as in the ganglion cell layer (Figure 3B).

**FIGURE 4:**
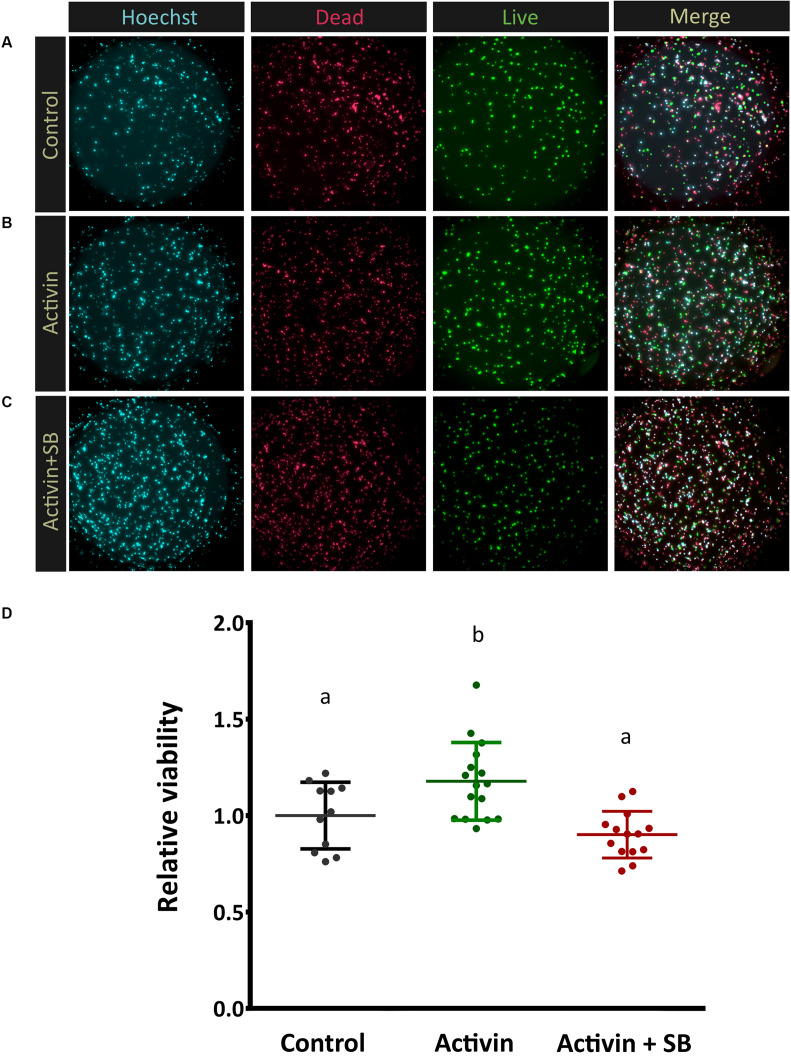
Activin signaling increases photoreceptor viability. In order to assess Activin A function *in vitro* enriched post-mitotic photoreceptor precursors were cultured alone **(A)**, in the presence of activin A only **(B)**, and with both activin A and the inhibitor SB-431542 **(C)** for 72 h. Representative images of live/dead cell staining (FDA, green, alive; PI, red, dead) are shown. Quantitative automated image analysis was carried out to determine the relative number of living cells **(D)** for each condition (*N* = 11, 16, 14 culture wells each for control, activin treated, and activin + SB groups, respectively). A median of 361 cells were counted per image (averaging 19, 000 total cells per treatment). Different letters represent a significant difference, *P* < 0.05.

With activin receptor expression confirmed at the protein level in photoreceptors, we sought to elucidate the role of activin signaling in these cells. Mouse retinas were dissociated, and photoreceptors were magnetically separated based on the expression of the photoreceptor-specific surface marker Cd73 (Koso et al., 2009; Eberle et al., 2011) and cultured in well plates in a minimal, defined media. Recombinant activin A was added at 10 ng/ml and cell viability was determined after 72 h in culture. Significantly more cells remained alive in activin-treated cultures compared to untreated controls (Figures 4A,B). As canonical Activin signaling involves a heterodimer of types 1 and 2 receptors, we treated parallel photoreceptor cultures with activin A in combination with the Tgfbr1/Acvr1b/Acvr1c inhibitor SB-431542 (Inman, 2002), which negated this beneficial effect (Figure 4C). Treatment of photoreceptor cultures with the Acvrl1/Acvr1/BmpR1a/BmpR1b inhibitor LDN 193189 (Sanvitale et al., 2013) did not attenuate the effect of activin A on photoreceptor survival (data not shown). This supports the hypothesis that activin A signaling in photoreceptors is mediated by canonical receptor signaling involving type 2 receptor complexes together with Acvr1b (Pangas and Woodruff, 2000).

**FIGURE 5:**
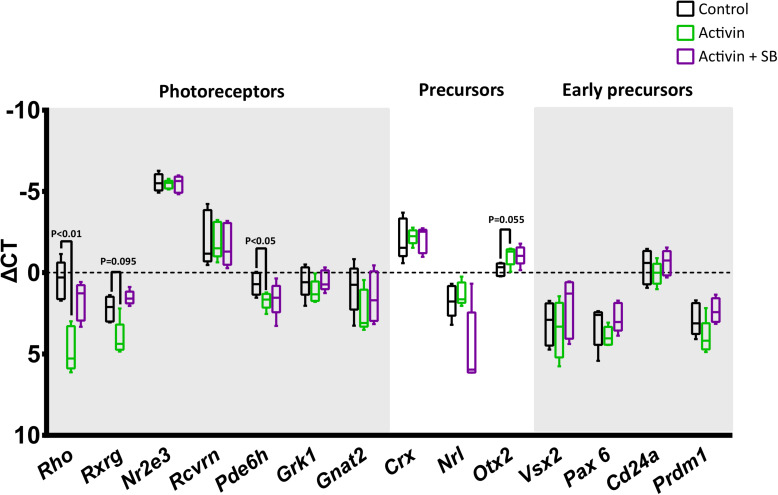
Activin A suppresses the transcription of mature photoreceptor genes. The impact of activin A signaling on transcription was assessed following 72 h culture without activin, with activin A only, and with activin A and the inhibitor SB-431542. While high levels of the photoreceptor-specific transcription factor Nr2e3 do not appear to be affected by activin signaling, the mature rod and cone markers Rho and Pde6h show statistically significant reductions. RT-qPCR data was normalized to three endogenous control genes (*Hprt*, *Polr2a*, and *Tbp*) and is shown as ΔCt plotted on a negative Y-axis (higher expression at the top).

Interestingly, while activin A signaling significantly increased survival in magnetically enriched photoreceptor cultures, subsequent immunostaining for the rod photoreceptor protein rhodopsin was negative (data not shown). To interrogate this finding further and assess whether the result was due to enhanced survival of some other cell type (which would be somewhat unexpected, as even pre-enrichment the retinal cell population is over 70% photoreceptors) (Akimoto et al., 2006), we analyzed these cultures by RT-qPCR. Intriguingly, while transcript levels of the photoreceptor-specific transcription factor Nr2e3 (Haider et al., 2000; Cheng et al., 2004; Peng et al., 2005) remained high, we observed significant decreases in the mature rod and cone markers Rho and Pde6h, respectively (Figure 5), which suggests substantial new areas for future investigation in light of the role for activin signaling in retinal development (see below).

## Discussion

Our receptoR tool allows for the interrogation of previously captured transcriptome data, pooled across multiple experiments and research groups, that can be arbitrarily organized to identify patterns and differential expression. It has been estimated that at least 10 expression sets are necessary to establish the profile of a tissue (Chang et al., 2011) and combining data from multiple laboratories has been shown to improve reproducibility of preclinical animal studies more effectively than increasing sample size alone (Voelkl et al., 2018). Our approach facilitates transcriptome analysis, taking into account both of these considerations, and provides for significantly more efficient use of scarce research resources when compared to the time and effort required to design and implement an experiment to obtain similar results.

Although RNA sequencing (RNA-seq) is rapidly entering widespread use for transcriptome profiling and has the ability to identify transcripts without *a priori* knowledge of them, the large body of microarray data accumulated over the past decades is still a tremendously valuable resource. Unlike most publicly available RNA-seq data, available in raw sequence formats that require alignment before meta analyses can be run (Lachmann et al., 2018), microarray probes are well-annotated and easily converted to gene expression information. Microarrays also offer a lower “barrier to entry” for researchers new to bioinformatics, particularly where the focus is on high-level behavior rather than rare transcripts or splice variants. The *a priori* design of a microarray experiment, while unsuited to the discovery of new transcripts, offers the ability to quickly parse expression data and filter for well-annotated RNA species. These in turn, more likely code for receptor proteins with known ligands. However, RNA-seq offers a much greater detection power, both in its range and freedom from fixed probes. Our approach, detailed here, should work equally well with any ‘omics data type, with adjustments for input signal type and corresponding annotations. We anticipate that ongoing developments in RNA-seq data processing (Bray et al., 2016) and decreasing costs of computing power will make on-the-fly RNA-seq processing of large numbers of datasets more practical for non-specialist laboratories in the near future, and plan to incorporate RNA-seq data analysis into future versions of receptoR.

While there are existing tools which make use of publicly available datasets stored on the GEO, to our knowledge receptoR is the first to allow users to assign arrays from multiple series records into customizable groups for downstream analysis (e.g., DEG prediction, cluster analysis). Recently, shinyGEO was developed with the purpose of examining gene expression and association with cancer survival (Dumas et al., 2016), but is limited to a single data series and is focused on gene association as opposed to gene discovery. Also making use of the shiny framework, Shiny Transcriptome Analysis Resource Tool (START) allows for visualization and analysis of RNA-seq data, however it does not query the GEO database (Nelson et al., 2017). Finally, GEO2R is GEO’s own tool and has related functionality in that groups can be redefined, and analysis undertaken on single GEO dataset. However, this analysis is not particularly straight forward for non-bioinformaticians as expression is restricted to probe-level data with several probes associated with one gene. Most importantly, GEO2R does not incorporate multiple series in the analysis.

We employed receptoR to generate hypotheses about cytokine receptor expression in mouse photoreceptors as compared to RPE to validate our bioinformatics approach. Of the multiple predictions, we chose to focus on activin signaling, confirming the presence of transcript (via RT-qPCR, Figure 2) and protein (via immunofluorescence, Figure 3). Given previous reports of a role for activin earlier in retinal development (Bertacchi et al., 2015), we assessed whether activin plays a role in photoreceptor survival. We determined that activin treatment significantly enhances the survival of enriched primary photoreceptor cultures, and that this effect is precluded by pharmacological inhibition of the activin type 1 receptor b (Acvr1b, also known as Alk4), consistent with previous reports that activin acts through SMAD2/3-mediated pathways with no activation of either ERK or AKT during photoreceptor differentiation from embryonic stem cells (Lu et al., 2017). This is not surprising as activin signaling has been long known for its neuroprotective effect (Kupershmidt et al., 2007).

To the best of our knowledge, this effect of activin signaling on nominally post-mitotic mammalian photoreceptor precursors has not been previously identified. However, activin signaling is known to play an important role in patterning the optic vesicle in early eye development. At that developmental stage, activin signaling promotes the expression of RPE-specific genes at the expense of retina-specific genes (Fuhrmann et al., 2000). Subsequently, activin signaling has been reported to promote cell cycle exit and differentiation into post-mitotic precursors in rodents (Davis et al., 2000). During directed differentiation from mouse embryonic stem cells to photoreceptor precursors, Lu and colleagues showed increasing levels of *Inhba*, *Acvr2a*, and *Acvr1b* throughout culture, while the addition of exogenous activin A upregulated *Otx2* and *Crx* (Lu et al., 2017), which is in line with our findings (potentially increased *Otx2* at *P* = 0.055, Figure 5). Our results showing that activin A-treated photoreceptors down regulate the rod photoreceptor markers *Rho* and *Pde6h* (Figure 5) (Swaroop et al., 2010; Brzezinski and Reh, 2015) likely reflect temporal changes in the role of activin signaling during development. Our findings are consistent with studies in chick retina cultures, where the role of activin during photoreceptor culture has been more extensively studied; treatment of photoreceptors with activin down regulates visual pigment genes including rhodopsin (Belecky-Adams et al., 1999; Bradford et al., 2005). Activin has also been reported to have an inhibitory effect on the differentiation of cultured chick photoreceptors (Belecky-Adams et al., 1999). The authors did not detect statistically significant increases in the number of live cells per dish in activin-treated groups, although the mean number of live cells was higher than controls at each time point from 48 h after plating onwards (Belecky-Adams et al., 1999). Our observations that activin represses later photoreceptor markers suggest regression to an earlier developmental stage and could hold potential as a new source of photoreceptors (Klassen et al., 2004), although significant further work will be required to confirm or refute this speculation.

In the nearer term, the ability of activin signaling to enhance photoreceptor survival has significant research potential, as the *in vitro* culture of primary photoreceptors is technically challenging, with the majority of cells dying shortly after initiating culture (LaVail et al., 1998; Traverso et al., 2003). This can be attributed to the fact that photoreceptors are very dependent on incompletely understood signals present in their niche, which are lost following their isolation. As a result, many *in vitro* studies of primary mouse photoreceptors use early post-natal photoreceptors that have exited mitosis and are undergoing maturation (Brzezinski and Reh, 2015; Unachukwu et al., 2016; Waldron et al., 2018). The inability to culture these cells efficiently has limited the ability of vision researchers to interrogate their behavior, and test the impact of various interventions such as neuroprotective molecules to combat retinal degeneration (Skaper, 2012). Importantly, the fact that our array data was pooled from tissues isolated at a range of ages (Supplementary Table 1) may have contributed to differences observed in gene expression between our array analysis and RT-qPCR (Figure 2). While receptoR is dependent on the data available, our results demonstrate that analysis of large publicly-available datasets can reveal important new biological information.

Our meta-analysis approach to expression analysis is related to those used extensively in cancer research, where diverse transcriptomics datasets can be brought together to identify mutations and chromosomal duplications that increase patient susceptibility (Newton and Wernisch, 2015). A similar approach has also been used to identify two genes that increase susceptibility to choroidal neovascularization in AMD, including a newly confirmed gene expressed in the retina (Akagi-Kurashige et al., 2015). Notably, Unachukwu et al. (2016) used the popular expression analysis software Ingenuity Pathway Analysis (IPA; Qiagen) to cross-match ligands present in the light-damaged retinal microenvironment with receptors expressed in photoreceptor precursors. While their work elegantly confirmed a known signaling mechanism (SDF-1α-CXCR4) involved in axon growth and guidance in photoreceptors, the IPA platform is closed and based on manually curated datasets (Unachukwu et al., 2016). Consequently, subscription fees make this a useful tool but not widely available. By contrast, the platform we present here makes use of publicly available datasets to generate hypotheses based on receptor signaling for non-experts in bioinformatics. Our tool allows for the straightforward analysis of cell communication pathways and makes use of multiple datasets to minimize laboratory or experimental bias.

While we cannot confirm the potential for activin signaling to affect photoreceptor differentiation, the identification of a new target to promote photoreceptor survival also has therapeutic implications, as photoreceptor degeneration is the leading cause of blindness in adults over 55 (de Jong, 2006). In dry age-related macular degeneration (AMD), photoreceptor loss is secondary to diseased and degenerated RPE and photoreceptor degeneration can be delayed by supplementing the retina with key RPE secreted factors (Tombran-Tink and Barnstable, 2003; Jayakody et al., 2015). It would be very interesting to test if supplementing the retina with activin A can delay photoreceptor degeneration in AMD animal models. A photoreceptor pro-survival factor could buy AMD patients time, reducing or delaying photoreceptor degeneration, and the activin signaling pathway has already been identified as a promising druggable target for other therapeutic indications (reviewed in Tsuchida et al., 2009). Such a factor could also be valuable in combination with cell-replacement therapies for diseased RPE (da Cruz et al., 2018). While the work presented here is an interesting first step, characterization of the mechanism by which activin improves photoreceptor survival warrants further investigation.

In summary, our receptoR tool was able to raise the specific, testable hypothesis that activin signaling is active in post-mitotic photoreceptors and/or maturing precursors, which we confirmed with subsequent experiments. In an era of restricted research resources, we hope to increase research efficiency by facilitating re-use of existing datasets by non-specialists. We anticipate this tool will be particularly useful for non-bioinformaticians wishing to mine transcriptome data to generate hypotheses regarding cytokine signaling in their cell type of interest. We have made the source code for receptoR freely available at https://github.com/derektoms/receptoR and a live version can be accessed at https://www.ucalgary.ca/ungrinlab/receptoR.

## Materials and Methods

### Bioinformatics

#### Querying Public Datasets

Our bioinformatics platform utilizes the open source software suite Bioconductor (Gentleman et al., 2004; Huber et al., 2015) based on the R programming language (R Development Core Team, 2008). Using the R package GEOQuery (Davis and Meltzer, 2007), we are able to import and process raw dataset files from the Gene Expression Omnibus (GEO).

To allow for straightforward integration of various datasets, the software makes use of only non-competitive (i.e., single color) arrays where each array contains a single biological sample that has been hybridized and its corresponding signals digitized. Data obtained from such arrays has been shown to be of the same quality as that obtained from two color, competitive arrays (Patterson et al., 2006). We also chose to initially limit microarray data to the two most common *in situ* oligonucleotide array platforms, ensuring consistency and simple quality control between experimental samples. This permits the pooling of multiple arrays, one per sample, as would be conducted in a wet lab microarray experiment. For mouse data, we use the GeneChip Mouse Genome 430 2.0 Array (GPL1260) platform, while human data is collected from the Affymetrix Human Genome U133 Plus 2.0 Array (GPL 570). As of December 2018, these two arrays contained 53 460 and 144 134 sample records, respectively.

#### Normalization and Differential Gene Expression

Retrieved expression data was then normalized using the log scale multi-array analysis (RMA) algorithm (Irizarry et al., 2003). Briefly, arrays were background corrected, normalized using quantile normalization, and log transformed. Following array normalization, we analyzed gene expression profiles among groups by fitting a multiple linear model based on probe level expression, with contrasts set between all our defined biological groups (Smyth, 2004). To predict DEGs between groups in a biologically meaningful way, we performed significance testing relative to a threshold, namely a log-fold change of greater than one (McCarthy and Smyth, 2009). We chose a lower threshold because the purpose of our application is an exploratory analysis of many expression datasets and a larger number of false positives was permissible given the requirement for external validation of these predictions.

Normalized expression data was analyzed by sparse partial least squares discriminate analysis (sPLS-DA) to determine membership for each observation of gene expression across all arrays (Lê Cao et al., 2011; González et al., 2012; Rohart et al., 2017). In other words, differences in expression in these genes were rated in terms of their abilities to discriminate groups. This allows for the identification and selection of relevant genes from each biological group.

#### Gene Ontology

The lists of receptor type were generated using KEGG and Panther annotations genes to ensure full coverage of biological pathways (Mi et al., 2010; Kanehisa et al., 2016) for both mouse and human genes, found in Supplementary Table S3. These gene lists are used to filter expression data to reduce the search to molecular receptors-coding genes.

Differential expression analysis was exported from receptoR, and used to generate a list of enriched gene ontology (GO) terms using GOrilla (Eden et al., 2007, 2009). All probes available on the microarray were used to generate enriched GO terms relating to biological function. From the input list, 8,266 of 8,478 gene terms were recognized, of which 5,024 had an associated GO term. All expression values and enrichment analysis are found in Supplementary Table S4. For visualization (Figure 2C), results were summarized by removing redundant GO terms by using REVIGO (Supek et al., 2011).

### Reverse Transcription Quantitative PCR (RT-qPCR)

RNA was isolated using the Norgen Total RNA Purification kit (Norgen Biotek cat. no. 37500) and quantified on an Implen spectrophotometer. Between 300 and 1000 ng of RNA was used for each reverse transcription reaction (iScript, BioRad cat. no. 1708841). PCR reactions were assembled using PowerUp SYBR Green Master Mix (Applied Biosystems, cat. no. A25742) and run on a Step One Plus Real-Time PCR System (Applied Biosystems). cDNA was tested using known reference primers before being used to quantitative experiments. Primers were also tested and found to have efficiencies of 100 ± 10%. Primer sequences are listed in Table 2. Values for non-detects were imputed from reaction values in the same biological category (e.g., other photoreceptor samples) (McCall et al., 2014) and relative expression (ΔCt) was calculated using an average of three stable endogenous controls: *Polr2a*, *Tbp*, *Hprt* (Vandesompele et al., 2002). Statistical differences were determined by a Mann–Whitney *U*-test.

**TABLE 2:** List of RT-qPCR primers.

**Gene symbol**	**Primers**		**Accession**
**Ackr3**	F	CACCGTCAGGAAGGCAAACC	NM_001271607.1
	R	AGAGTGATTTGTGGGGTGTCC	
**Acvr2a**	F	GCGTTCGCCGTCTTTCTTATC	NM_007396.4
	R	GTTGGTTCTGTCTCTTTCCCAAT	
**Acvr2b**	F	AGGCAACTTCTGCAACGAG	NM_007397.3
	R	CTTCCGATGACGATACATCCAG	
**Atp5a1**	F	CCTTGACCTTCCTTTGCGCT	NM_007505.2
	R	GCACCAACAAAGGATGACCC	
**Cd24a**	F	TTCTGGCACTGCTCCTACCC	NM_009846.2
	R	CTGGTTACCGGGAAACGGT	
**Crx**	F	CCAGTACCTGAACATCCAGGAG	NM_007770.4
	R	GGGCACTTGAGTATGGGACAG	
**Cxcr4**	F	CCATGGAACCGATCAGTGTGA	NM_001356509.1
	R	TCCATTGCCGACTATGCCAG	
**Gnat2**	F	AGTCTCAAGGCAAGATAGGAAAA	NM_008141.3
	R	ACTGATGCCACTCCCCATTT	
**Gpr75**	F	CTCAGGCTTCGTCATCATGTC	NM_175490.4
	R	AGGGTAAGGAGCAAGATGCAG	
**Grk1**	F	GGGACCCCAGGTTTCATGG	NM_011881.3
	R	GGCTGCGATCATCTCGTACA	
**Hprt**	F	GCAAACTTTGCTTTCCCTGATT	NM_013556.2
	R	CAAGGGCATATCCAACAACA	
**Kdr**	F	TTTGGCAAATACAACCCTTCAGA	NM_001363216.1
	R	GCAGAAGATACTGTCACCACC	
**Ncam2**	F	CTGCTCGGGTTGCTTGTCA	NM_001113208.1
	R	CCCACACTAAGCTCTACTTTGCT	
**Nr2e3**	F	CAGCATAGCAAGGCTCACCA	NM_013708.4
	R	ACCTCAAAGATGGGAGCAGG	
**Nrl**	F	CCGTCTGGGAATGAGCGAG	NM_001271916.1
	R	GGCTGGTGTCGTCCCTTTT	
**Otx2**	F	CAAATCTCCCTGAGAGCGGA	NM_001286481.1
	R	AGGGTCCTTGGTGGGTAGAT	
**Pax6**	F	CGGCTTTGAGAAGTGTGGGA	NM_001244198.2
	R	CGGCTTTGAGAAGTGTGGGA	
**Pde6h**	F	AGCGACTAGACAACTTACGGG	NM_023898.4
	R	GTGCTTTGCTTTCAGGCACG	
**Plxna2**	F	AACCTGTCTGTGGTTCTGCTC	NM_008882.2
	R	TCCAGTCACGATTCTCAGAGT	
**Polr2a**	F	TGTGCAGGAAACATGACCGA	NM_001291068.1
	R	GAAGCAGACACAGCGCAAAA	
**Prdm1**	F	CCCGCGGCCGTAGAAAA	NM_007548.4
	R	CCAGTCTCTGCCAGTCCTTG	
**Rcvrn**	F	AGTGGGCCTTCTCGCTCTA	NM_009038.2
	R	TCATTTTGAAGATAGCCATGACG	
**Rho**	F	CGCACACCCCTCAACTACAT	NM_145383.2
	R	CAGGGCGATTTCACCTCCA	
**Robo3**	F	AGATGAACTTGTTCGCGGACT	NM_001164767.1
	R	GGAAGCAGACTAGGGTTGAGC	
**Rxrg**	F	GAAGCGCAGCAGAGGAATGA	NM_009107.3
	R	CAAGGCTACTGAAGGGCTCA	
**Tbp**	F	ACCGTGAATCTTGGCTGTAAAC	NM_013684.3
	R	ACCGTGAATCTTGGCTGTAAAC	
**Thrb2**	F	CTGGGCAGTGAATCAGCCTTA	NM_009380.3
	R	GTCCCCACACACTACACAGAG	
**Vsx2**	F	CAAGAAGCGTAAGAAGCGGC	NM_001301427.1
	R	AGACATCTGGGTAGTGGGCT	

### Mouse Retina Dissection, Photoreceptor Precursor Enrichment, and Culture

All experiments involving animals were carried out in accordance with the recommendations of the Canadian Council on Animal Care’s “Guide to the Care and Use of Experimental Animals.” The protocol was approved by the Animal Care Committee at the University of Calgary. Retinal tissues were accessed under a secondary-use protocol, from animals freshly euthanized as controls in other experiments in neighboring laboratories, where the retinal tissue would otherwise be discarded. Many tissue types can be easily and regularly obtained in this way, and we encourage researchers to assess how these might reduce their own need for animals, and the associated costs. Retinas from euthanized mice at post-natal day (PN)4 were dissected from the eyes and dissociated in DPBS without calcium or magnesium (DPBS) containing 0.125% trypsin (Sigma cat. no. T1005) and 0.3 mg/ml DNaseI (EMD Millipore cat. no. 260913) for 4–6 min in a shaking waterbath at 37°C (*f* = 120 rpm). The enzymatic solution was stopped by adding an equal volume of DPBS containing 20% FBS. The cell suspension was triturated with a fire-polished glass pasteur pipette before being spun at 333 rcf for 5 min. Supernatant was removed and cells were resuspended in 500 μl EasySep buffer (StemCell cat. no. 20144) before being magnetically enriched on an EasySep system using the Mouse PE Positive Selection kit (StemCell cat. no. 18554) according to manufacturer’s directions. Anti-CD73 antibody conjugated to PE (BD cat. no. 550741) was used at 3 μg/ml to select a positive photoreceptor fraction.

Prior to culture, black-walled 96 well plates (Grenier cat. no. 655090) were coated with 50 μg/ml poly-D-lysine (Corning cat no. 354210) for at least an hour before being washed twice with sterile distilled water and allowed to dry. Photoreceptors were plated at a density of 3.1 × 10^5^ cells/cm^2^ in a volume of 200 μl per well (media depth of 6 mm), and cultured in DMEM/F12 (70:30) supplemented with 2% B-27 and 1% antibiotic-antimycotic (all Thermo/Gibco cat. no. 11965, 11765, 17504, 15240).

### Photoreceptor Viability Assay

After 72 h in culture, photoreceptor media was replaced with DPBS containing 5 μg/ml Hoechst 33342 (Invitrogen cat. no. H21492), 50 ng/ml fluorescein diacetate (FDA; Sigma cat. no. F7378), and 2.5 μg/ml propidium iodide (PI; Sigma cat. no. P4170) to detect nuclei, live and dead cells, respectively. After 5 min incubation at 37°C, this staining solution was removed and the cells were washed with DPBS. Plates were imaged on an Olympus IX83 Microscope at 200X magnification using MicroManager software (Edelstein et al., 2014). Four or five non-overlapping images per well were taken.

Images were processed using a Cell Profiler pipeline that assessed co-localization of a nucleus with either the live or dead stains to determine the percentage of cells alive (Carpenter et al., 2006). Images were pre-processed using ImageJ by subtracting the background from the captured images. Primary objects were identified and subsequently related to establish a parent–child relationship between blue–red objects and blue–green objects. Finally, the filter objects module was used to quantify the number of colocalized objects that were both blue and red, and objects that were blue and green, as the blue–red objects were considered “dead” cells and the blue–green objects were considered “live” cells. Viability was calculated as the number of “live” cells divided by the sum of “live” and “dead” cells.

Results from all images of a single well were averaged and this value represents a biological replicate. Experiments were repeated three times with four litters of mice, representing a total *N* = 11, 16, 14 culture wells each for control, activin treated, and activin + SB groups, respectively. Differences in viability between groups were determined using a one-way ANOVA test followed by a Tukey Honest Significant Difference test.

### Immunostaining

Whole eye sections were prepared by fixing dissected PN4 mouse eyes with the RPE removed in 4% paraformaldehyde (PFA) in DPBS overnight at 4°C. Fixed eyes were then transferred to a 15% sucrose solution for 24 h and a 30% sucrose solution for 24 h at 4°C. Cryoprotected eyes were then embedded in clear frozen section compound (VWR cat. no. 95057-838) compound before being frozen in a dry ice and 2-propanol slurry. Slides were prepared by cutting 20 μm sections and mounting them to charged slides. The following antibodies were used to detect the various epitopes: activin receptor type 2A (Abcam, cat. no. ab96793), Activin receptor type 2B (Abcam, cat. no. ab76940), OTX2 (Abcam, cat. no. ab114138), and Rhodopsin (Abcam, cat. no. 98887).

Cultured photoreceptors were fixed by adding 100 μl of 4% PFA in DPBS to each well, and incubated for 5 min at room temperature (RT; 22°C). The wells were then washed three times with DPBS. Photoreceptors were permeabilized by incubating each well with 100 μl of 0.1% Triton X-100 (Amresco cat. no. M143) for 5 min at RT, followed by three more washes in DPBS. Each well was blocked with 1% (w/v) bovine serum albumin (Sigma cat. no. A3294) in DPBS for 10 min at RT followed by another three washes. Primary antibodies were then added to the samples (1/500) in 0.5% BSA in DPBS (100 μl/well). Primary antibody is incubated over night at 4°C. Samples were then washed three times in DPBS and blocked with 1% BSA for 10 min at room temperature. Secondary antibody was then added (1/1000) in 0.1% BSA (100 ul/well) and incubated for 1 h at RT. Cells were then washed (3X) with DPBS and stained with 100 ul of (1/2000) 4′,6-diamidino-2-phenylindole (DAPI; Thermo Fisher Scientific cat. no. D1306) solution for 5 min at room temperature. The samples were then washed (3X) with PBS and stored in the dark at 4°C until they were imaged.

## Data Availability Statement

The datasets analyzed for this study can be found in the GEO database(https://www.ncbi.nlm.nih.gov/geo/), using GSM accession numbers detailed in Supplementary Table S1.

## Ethics Statement

This study was carried out in accordance with the recommendations of the Canadian Council on Animal Care’s Guide to the Care and Use of Experimental Animals. The protocol was approved by the Animal Care Committee at the University of Calgary.

## Author Contributions

DT, AA-A, and MU designed the experiments and interpreted the results. DT and AA-A wrote the manuscript. AA-A, DT, SS, and QT conducted the biological validation experiments and analyzed the data. DT and MW developed the web tool. AA-A and DT contributed to the mathematical and statistical methods. MU originated the concept and edited the manuscript. All authors reviewed and approved the final manuscript.

## Conflict of Interest

The authors declare that the research was conducted in the absence of any commercial or financial relationships that could be construed as a potential conflict of interest.

## Supplementary Material

TABLE S1 Annotation and accession information for all datasets used in this study.

TABLE S2 Differentially expressed gene lists.

TABLE S3 Receptor type gene lists.

TABLE S4 GO enrichment analysis.

METHODS S1 Step-by-step instructions for using the online version of receptoR.
