# Supplementary material for: Automated Hypothesis Generation to Identify Signals Relevant in the Development of Mammalian Cell and Tissue Bioprocesses, With Validation in a Retinal Culture System
Source: Front Bioeng Biotechnol. 2020 Jun 4;8:534. doi: 10.3389/fbioe.2020.00534 (PMC7287043; doi:10.3389/fbioe.2020.00534)
Supplement: METHODS S1 — Step-by-step instructions for using the online version of receptoR. [file Data_Sheet_1.docx]

Supplementary Methods: Using receptoR

# Getting Started

Connect to the web application at <https://www.ucalgary.ca/ungrinlab/receptoR>. If you are starting a new analysis, click the “Search Transcriptome Database” tab at the top of the screen; if you want to begin with a processed dataset, click the “Load Expression Datasets” tab and jump to step 3, below.

# Building an Expression Dataset

In order to combine public expression data into a single dataset, there are three steps: 1) searching for and adding arrays; 2) assigning each array into one of three user-defined categories; and 3) downloading and processing the raw data for downstream analysis.

## Searching for Arrays

Currently, receptoR uses Affymetrix arrays based on either human or mouse expression sequences. Choose your desired species and then enter search terms in the box below. Boolean operators (AND, OR, NOT) are permitted but need to be capitalized to function correctly. Search results will be displayed on the right side of the screen. To add arrays to your analysis, select the appropriate row/s and click the “Add array to experiment” button. This will bring you to the “Assign” sub-tab, detailed below.

## Assigning Arrays to Categories

Once arrays are added to the analysis, they need to be categorized for differential gene expression analysis. Enter names for each category in the side panel at left and use the “Select a Category” dropdown and “Assign” button to assign each array (rows in the main panel) to a category. The background colour of the row will change to reflect its category assignment. Columns can be hidden or shown to get more information about each array using the “Column visibility” button.

Continue adding and assigning arrays by repeating steps 2.1 and 2.2.

## Processing Array Data

When a sufficient number of arrays have been added (typically > 10 per group), the “Process” sub-tab confirms array selection and assignment, and allows you to enter a dataset name and any additional comments. Click the “Download Report” button to keep a copy of this data (CSV format) and then click the “Process” button to download and process raw expression data.

# Loading Gene Expression Data

To begin analysis, select an experiment for analysis from the dropdown menu. This can be the experiment you just created in step 2, or a previously created experiment. When this loads, a checkbox list will appear prompting you to select one or more receptor types to analyze (e.g. cytokine receptors, nuclear hormone receptors). Additional genes can be added by entering their gene symbol in the text box. All differentially expressed gene data can be downloaded by clicking the “Download differential gene expression analysis” button for downstream processing in other applications.

When gene lists have been selected, all member genes are shown in the “Gene-by-gene Expression sub-tab, at right. Clicking on any gene will bring up a violin plot showing the distribution of expression values across arrays and between samples. The “Quality control” sub-tab lets you see information on array normalization and RNA degradation. At least one receptor gene set needs to be selected for further analysis.

# Expression analysis

The final two tabs, “Gene-level Expression” and “Sample-level Expression”, are used to explore the data. Clustered heatmaps of gene expression, boxplots by gene and boxplots by category allow you to identify significantly different receptor genes between categories. A minimum of 10 genes are needed for several of these analyses, so parameter tuning (e.g. category comparisons shown, differential genes only) may be necessary from the side panel at left.

Discriminant analysis and component loadings, found under the “Sample-level Expression” tab, shows a higher level view of the expression data including genes that contribute most significantly to variability between categories.
